# Progress in heart failure management in the Netherlands and beyond: long-term commitment to deliver high-quality research and patient care

**DOI:** 10.1007/s12471-020-01453-7

**Published:** 2020-08-11

**Authors:** L. M. G. Meems, D. J. van Veldhuisen, R. A. de Boer

**Affiliations:** Department of Cardiology, University Medical Center Groningen, University of Groningen, Groningen, The Netherlands

**Keywords:** Heart failure, The Netherlands, SGLT2 inhibitors, Nutrition, Diuretics, Home monitoring

## Abstract

Heart failure (HF) remains a major global problem. In the Netherlands, 1.5–2.0% of the total population is diagnosed with HF. Over 30,000 HF patients are admitted annually in the Netherlands, and this number is expected to further increase given the ageing population and the chronic nature of HF. Despite ongoing efforts to reduce the burden of HF, morbidity and mortality rates of this disease remain high. However, several new treatment modalities have become available or are expected to become available in the coming years. This review will provide an overview of HF research conducted in the Netherlands (often in an international setting) that may have clinical consequences for diagnosis, treatment and prevention of HF, and will also evaluate outcomes of larger clinical trials that have been conducted in the Netherlands.

## Dutch contribution to the field

- Multicentre HF research has a long history in the Netherlands with the first trial in 1993.
- Dutch institutions today still have a leading role in various national and international multicentre studies.
- Dutch HF research and patient care covers a broad field and includes a variety of aspects that may contribute to improvement and optimisation of HF prevention, disease management and treatment.

## Introduction

Heart failure (HF) remains a major and global problem [1]. In Western European countries such as the Netherlands, 1.5–2.0% of the total population is diagnosed with HF. In 2018, approximately 250,000 patients in the Netherlands were living with HF. They presented with a high admission rate of more than 16,000 admissions for HF per year for men (a 2% increase compared with 2017) and more than 14,000 per year for women (a 10% increase compared with 2017) with an average hospital stay of 7 days [2]. So, clearly, HF is a significant burden to patients and healthcare systems.

HF can be subdivided into three different subtypes: HF with reduced ejection fraction (HFrEF), HF with mid-range ejection fraction (HFmREF) and HF with preserved ejection fraction (HFpEF) [1]. Signs and symptoms of these subtypes are quite similar, but underlying pathophysiology may differ substantially. Despite ongoing efforts to reduce the burden of this disease, HF morbidity and mortality rates remain high [1]. Progress has been made in reducing morbidity and mortality rates in patients with chronic HFrEF due to the introduction of beta-blockers [1], angiotensin-converting-enzyme (ACE) inhibitors [1], angiotensin II receptor blockers (ARB) [1], mineralocorticoid antagonists (MRA) [3], and more recently due to the discovery of the angiotensin receptor-neprilysin inhibitor (ARNi) sacubitril-valsartan [4]. Although these therapies have improved quality of life and survival in patients with chronic HFrEF, a challenge remains in the treatment of HFpEF and acute HF, for which treatment is still lacking [5]. A continuous and substantial effort is being made to develop novel drugs that further improve mortality and morbidity rates in patients with all types of HF, both acute and chronic.

High-quality, multicentre HF research has a long history in the Netherlands. The first multicentre HF trial was conducted 30 years ago and was published in 1993 [6]. This was a collaboration between academic cardiology centres, and a conglomerate of larger non-academic sites, who had an interest in performing cardiovascular clinical trials, the *Werkgroep Cardiologische Centra Nederland *(WCN). This connection has proven to be very successful ever since. In many HF trials, the Netherlands was always among the top-enrolling countries [7, 8] and the network was also active in other fields of cardiovascular disease [9]. These early efforts created a strong situation for today where Dutch institutions are leading in several multicentre national and international studies. For example, Dutch HF research has played a pivotal role in understanding how HF pathophysiology is influenced by other cardiovascular diseases, such as atrial fibrillation [10–12], and other non-cardiovascular comorbidities, such as decreased kidney function [13], and more recently oncology [14].

The scope of this review is to provide an overview of contemporary HF research conducted in the Netherlands that may have clinical consequences for the diagnosis, treatment and prevention of HF, but will also evaluate outcomes of recent larger clinical trials in which the Dutch contribution has played an important role.

## Improving HF outcomes: from beta-blocker to ARNi to SGLT2 inhibitor

The introduction of beta-blockers—already over 20 years ago—has improved HF survival rates in patients with chronic HF, with a substantial reduction in mortality of 35% [15–17]. Ever since, a continuous effort is being made to further improve mortality and morbidity rates in patients with HF. Over the last years, several international and multicentre clinical trials, including various Dutch sites, were conducted to evaluate the effect of two groups of potential novel HF therapeutics: namely the ARNi sacubitril-valsartan and sodium-glucose cotransporters (SGLT2) inhibitors.

## Angiotensin receptor-neprilysin inhibitors in HF

In 2014, the results from the PARADIGM-HF study caused a shift in HF care as it appeared that treatment with the combination of ARB and a neprilysin inhibitor (ARNi, sacubitril-valsartan), instead of the gold standard (the ACE inhibitor enalapril), significantly lowered the composite endpoint cardiovascular (CV) mortality and HF hospitalisation for patients with HFrEF [4]. After this trial, the ARNi was proposed as standard care for patients with an ejection fraction <40%, first after optimal titration with an ACE inhibitor, but now also more upfront, as first-line therapy. It, however, remained unknown if this drug would also exert beneficial effects in patients with HFpEF. The PARAGON-HF (Prospective Comparison of ARNi with ARB Global Outcomes in HFpEF) trial aimed to evaluate the effect of the ARNi sacubitril-valsartan in patients with HFpEF [18]. PARAGON-HF enrolled 4679 typical and symptomatic HFpEF patients, but treatment with sacubitril-valsartan did not significantly improve CV mortality and HF hospitalisations. Subgroup analysis revealed a suggestive benefit of sacubitril-valsartan therapy over valsartan monotherapy in patients with an ejection fraction in the lower range (45–57%) (rate ratio 0.78, confidence interval (CI) 0.64–0.95), and in women (rate ratio 0.73, CI 0.59–0.90). Additional studies will be required to evaluate whether treatment with sacubitril-valsartan will be beneficial in these specific subgroups.

Since ARNi therapy was predominantly started in stable patients because of fear of hypotension, the PIONEER-HF (Comparison of Sacubitril-Valsartan versus Enalapril on Effect on NT-proBNP in Patients Stabilised from an Acute Heart Failure Episode) evaluated the efficacy and safety in unstable patients, who were hospitalised for acute decompensated heart failure (ADHF) [19]. A total of 881 patients, who had to be haemodynamically stabilised, were randomly assigned to receive sacubitril-valsartan (target dose 97–103 mg twice daily) or valsartan (160 mg, twice daily) during a period of 8 weeks. The 440 patients who received sacubitril-valsartan showed a greater reduction in NT-proBNP levels already from week 1 on (ratio of change 0.76; 95% CI 0.69 to 0.85), while safety outcomes (incidence of worsening renal function, hyperkalaemia, symptomatic hypotension and angio-oedema) did not differ between the groups. Furthermore, an exploratory analysis also showed that in-hospital initiation of sacubitril-valsartan was associated with a lower rehospitalisation rate for HF at 8 weeks. Overall, this study confirmed that it is safe to start sacubitril-valsartan therapy in patients hospitalised for ADHF after haemodynamic stabilisation and that is lowers NT-proBNP levels almost directly after therapy is initiated. Ideally, more studies should be conducted to further evaluate the potential beneficial effect sacubitril-valsartan may have on preventing rehospitalisation due to ADHF.

## Sodium-glucose cotransporters (SGLT2) inhibitors in HF

SGLT2 inhibitors have successfully prevented new-onset HF in individuals without HF [20], alongside a reduction of CV mortality and major CV events. However, it remained unclear if these glucose-lowering drugs also exert beneficial effects in individuals with *prevalent* HF. The DAPA-HF (Dapagliflozin and Prevention of Adverse Outcomes in Heart Failure) trial was designed to evaluate the efficacy and safety of the SGLT2 inhibitor dapagliflozin in patients with HFrEF, regardless of the presence or absence of diabetes [21–23].

**Fig. 1:**
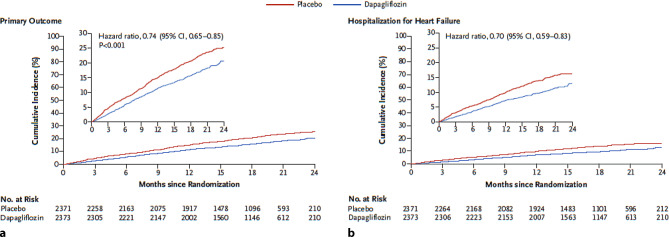
Kaplan-Meijer curves of the effect of treatment with dapagliflozin on top of standardised HF care on: **a** Primary outcome; **b** Hospitalisation for heart failure (reproduced with permission) [24]

The results of DAPA-HF were striking (Fig. 1). Treatment with dapagliflozin reduced the primary composite outcome of CV death or worsening HF (hospitalisation or an urgent visit resulting in intravenous therapy for HF) (386 patients vs. 502 patients; hazard ratio (HR), 0.74; 95% CI 0.65 to 0.85; *p* < 0.001). Patients receiving dapagliflozin also experienced less HF symptoms, and the beneficial effects were considered substantial and clinically meaningful. These benefits occurred shortly after dapagliflozin treatment was started, and were observed in HFrEF patients with and without type 2 diabetes mellitus [24]. There was no excess of serious adverse events in the dapagliflozin group, and renal endpoints were numerically even less frequent, suggesting that the SGLT2 inhibitor dapagliflozin combines efficacy with safety. This implies that dapagliflozin may be added to standard HF care in order to improve the mortality and morbidity rate in chronic HFrEF patients.

The EMPA-RESPONSE-AHF, an investigator initiated trial conducted in the Netherlands, was a randomised, placebo-controlled, double-blind, multicentre pilot study that evaluated the effect of another SGLT2 inhibitor, empagliflozin (10 mg/day) in ADHF patients with and without diabetes [25]. Its primary outcomes were change in visual analogue scale (VAS) dyspnoea score, diuretic response, change in NT-proBNP levels and length of hospital stay. Treatment with empagliflozin did not improve the primary endpoint: no change in VAS dyspnoea score, diuretic response, length of stay, or change in NT-proBNP levels was observed. Empagliflozin, however, improved secondary endpoints: it increased urinary output up to day 4 of hospitalisation and successfully reduced a combined endpoint of in-hospital worsening HF, rehospitalisation for HF or death at 60 days after enrolment when compared with placebo (4 (10%) vs. 12 (33%), *p* = 0.014). Treatment with empagliflozin appeared to be safe, well tolerated and without adverse effects on blood pressure or renal function. Overall, this trial suggests that treatment with empagliflozin is, at least, safe in patients with ADHF, but clearly larger randomised trials are needed.

These first SGLT2 inhibitor studies in HF suggest that treatment with these drugs in safe and well-tolerated and that it may also improve cardiovascular outcomes in HFrEF patients. However, much remains unknown and the on-going multicentre randomised controlled trials, in which also many Dutch hospitals and patients are participating or have participated, will hopefully provide sufficient knowledge as to whether SGLT2 inhibitors should be added as novel therapeutics to the HF-arsenal for HFrEF, HFpEF or ADHF.

## Vericiguat, a cyclic guanosine monophosphate donor, may improve HF hospitalisation rates in HFrEF

Very recently, the VICTORIA (Study of Vericiguat in Participants with Heart Failure with Reduced Ejection Fraction) study was published [26]. Vericiguat, a novel oral soluble guanylate cyclase stimulator, enhances the cyclic guanosine monophosphate pathway by directly stimulating soluble guanylate cyclase and by sensitising soluble guanylate cyclase to endogenous nitric oxide. VICTORIA was a phase 3 trial and randomised 5050 patients with chronic HFrEF (with a high percentage in New York Heart Association class III and IV) to receive vericiguat or placebo. The primary outcome was a composite of death from cardiovascular causes or first hospitalisation for HF and after a median of 10.8 months the primary outcome was reduced by 10% by vericiguat (HR 0.90; 95% CI 0.82 to 0.98; *p* = 0.02). This was explained by less patients hospitalised for HF (HR 0.90; 95% CI 0.81 to 1.00). CV death was not reduced (HR 0.93; 95% CI 0.81 to 1.06). It is unclear where vericiguat will be positioned in the HFrEF treatment algorithm.

## Biomarker-guided therapy as guidance to improve HF outcomes

Natriuretic peptides, and especially NT-proBNP, have an important role when it comes to HF diagnosis. According to current guidelines, the role of NT-proBNP in disease management is limited, controversial and it should not be used as such [27, 28]. Guidelines, however, do not provide recommendations for NT-proBNP guided ADHF disease management and the PRIMA II Trial (Can NT-proBNP-Guided Therapy During Hospital Admission for Acute Decompensated Heart Failure Reduce Mortality and Readmission?) was designed to prospectively evaluate the effect of NT-proBNP guided therapy in patients hospitalised for ADHF and NT-proBNP levels >1700 ng/l. Although patients in the NT-proBNP-guided therapy group were discharged with significantly lower NT-proBNP levels (reduction of >30% in 80%, vs. 64% in control group, *p* = 0.001), this reduction in NT-proBNP did not improve mortality rates or the number of HF readmissions at 3 months or at 6 months, suggesting that NT-proBNP guided therapy does not have additional value to standardised care [29]. Interestingly, in a substudy, it was confirmed that NT-proBNP levels at discharge had similar predictive outcome values for HFrEF and HFpEF patients, regardless of reduction in NT-proBNP levels [30]. Therefore, the role for NT-proBNP to improve HF outcomes does not seem to differ between ADHF and chronic HF, and may only be useful in diagnosing and not so much in predicting and evaluating disease management in patients with ADHF.

## Refined approach of a well-known HF therapy: tailored-diuretic therapy

Episodes of ADHF are associated with increased mortality and morbidity rates [31]. Diuretics are a cornerstone in HF therapy and are well-known and frequently used in ADHF patients to reach a euvolaemic state [32]. In a recent statement paper, supported by the Heart Failure Association (HFA) of the European Society of Cardiology (ESC-HF) [33], a novel approach has been proposed that uses a more personalised approach to assess and evaluate the success of diuretic therapy. Several Dutch colleagues were involved in the development of this algorithm. In short, prior to therapeutic intervention, one has to evaluate if a patient is diuretic naïve or not: diuretic naïve patients can start with a low-dose intravenous (IV) loop diuretic (such as 20–40 mg furosemide IV), while non-diuretic naïve patients need an IV starting dose that is 1–2 times the 24-hour oral dose. It is advised to assess spot urinary sodium levels after 2 h of treatment to evaluate the success of diuretic therapy. After 6 h, the total urinary output should be assessed, and based upon spot urinary sodium levels and urinary production diuretic therapy can be continued or requires doubling of the administered dose. If persistent congestion remains on the second day of admission, the 24-hour urine output must be evaluated: when urinary output is below 3–4 litres, the dose of the loop diuretics should be doubled until the maximal dose is reached. If the maximal loop diuretic dose has been reached and diuresis is still <100 ml/h, a combination diuretic therapy needs to be considered by adding thiazides (first line), acetazolamide or amiloride (second line) or SGLT2i (third line) to the maximal dose of loop diuretics. If congestion still remains, diuretic therapy is claimed to be unsuccessful and ultrafiltration should be considered.

A great deal of patients are discharged with residual clinical congestion [31]. Preparation for discharge is therefore of great importance in the final stage of hospitalisation: patients should be clinically stable on oral medication for at least 24 h before they can be considered for discharge. Also, after they are discharged a multidisciplinary program with early ambulatory clinical and laboratory follow-up is required to reduce readmission and improve quality and longevity of life.

This position paper provides a hands-on strategy on how to treat patients with ADHF, and aims to optimise patient-based HF care. Caveats are the lack of prospective trial data, especially in the difficult to treat patients with diuretic resistance, where most recommendations are opinion based. The use of inotropes remains controversial in the absence of trial data, but for refractory patients they are widely used. The position statement calls for trials to be conducted in this complex patient category.

## HF disease management: attention for nutritional deficiencies

HF disease management has an important role in HF care in the Netherlands, and HF disease management programs are nationally implemented. These programs oftentimes provide intensive support by a specialised nurse under the supervision of a cardiologist. Already in 2008 it became clear that this disease management strategy was safe, even though it was not associated with a reduction in mortality and hospitalisation rates [34]. Ever since, HF disease management programs have been further developed and specialised nurses guide patients with HF by providing medical advice regarding volume homeostasis and use of diuretics, but also focus on lifestyle advice including nutrition.

Nutritional deficiencies are common in HF patients, and this topic remains a continuous line for research. For example, vitamin D deficiency was found to be associated with poor outcome in HF patients [35], but later research revealed that a low vitamin D status was not associated with risk of developing HF [36]. Long-term supplementation of vitamin D in HF patients did not reduce mortality in HF patients [37] and a widespread use of vitamin D supplements for patients with HF can no longer be advocated [38]. The sequelae in the vitamin D studies once again demonstrate that associative studies should not replace randomised controlled trials, which prospectively address whether restoring nutritional deficiencies with supplementation is beneficial.

Another common deficiency is iron deficiency: half of patients with HF have iron deficiency, which is known to negatively impact symptoms and is associated with increased mortality and worse prognosis in HF patients. Its aetiology is not yet fully understood, but it is considered to be multifactorial and results from reduced iron uptake, impaired iron storage and increased iron loss [39]. Oral supplementation of iron in HF patients was not effective, and was actually associated with a higher incidence of adverse effects in a large proportion of patients (up to 40%) [40]. Several clinical trials have evaluated the effect of intravenous iron supplementation by ferric carboxymaltose in symptomatic iron-deficient chronic HF patients and observed that IV treatment restored iron stores and improved symptoms and quality of life [41, 42]. It is currently being investigated whether IV iron also reduces cardiovascular mortality and recurrent hospitalisations in iron-deficient ADHF patients (AFFIRM-AHF, (ClinicalTrials.gov NCT02937454), with important Dutch contributions) [43]. To date, the European Society of Cardiology (ESC) HF guidelines advocate testing all HF patients for anaemia and iron deficiency (including serum ferritin and transferrin saturations), and recommend treatment with IV ferric carboxymaltose (Class IIA, level of Evidence A recommendation) for symptomatic HF patients with iron deficiency to improve HF symptoms and quality of life [1].

## Future perspectives: towards personalised medicine

More and more attention is drawn to personalised medicine to optimise disease prevention and disease management. Sex-specific differences are increasingly recognised as potential targets to improve personalised HF care. For example, in a current review from Suthahar et al. it was demonstrated that key HF biomarkers display sex-related differences, and that the clinical meaning of these differences is not clear yet. To date, studies do not recognise the importance of sex-specific evaluation of biomarker levels. However, an adapted strategy that examines biomarker levels in men and women separately may reveal important sex-related differences that will contribute to improved HF care [44].

Men and women may also require sex-specific therapeutic strategies. Pharmacokinetics are different between men and women due to differences in fat distribution, body weight and plasma volume, while haemodynamic effects may also exert sex-specific properties due to sex-specific differences in cardiac output, hepatic flow and glomerular filtration rate [45–48]. So far, guideline-recommended strategies are similar for men and women with HFrEF, and guidelines have ignored the importance sex may have, in line with the lack of data. Previous pharmacological studies indicated that with the same dose, maximum concentrations of ACE inhibitors, beta-blockers and ARBs were 2.5 times as high in women compared with men [45, 49, 50]. In a recent post-hoc analysis, Santema et al. evaluated recommended doses of HF therapy and their effect on hospitalisation rates and observed a distinct sex-specific effect: while lowest hazard ratios for hospitalisation were observed at 100% of recommended doses of ARBs, ACE inhibitors and beta-blockers in men, women already showed a 30% risk reduction at 50% of recommended treatment doses, without further risk reduction at higher dose levels [51]. Future studies are eagerly awaited to answer the question whether optimal therapy is the same for men and women.

Further optimisation of personalised medicine might also be obtained by reconsideration or repurposing of well-known drugs, such as digoxin [52]. Current recommendations on the use of digoxin in HF are made on outcomes from a multicentre trial in 1997 [53] in an era that many of the currently used HF therapeutics did not even exist. Two large randomised controlled trials (EudraCT: 2013-005326-38, DECISION, and ClinicalTrials.gov Identifier: NCT03783429) in patients with HF will re-evaluate the role of digoxin in modern HF treatment. DECISION will also include a significant proportion of patients with atrial fibrillation (AF); this combination of AF and HF has not yet been explored in digoxin-related research.

In addition, AF is increasingly recognised as an important player in HF. Especially in HFpEF, detection of AF is important, as it appeared highly predictive of underlying HFpEF [54, 55]. The development of the recent American H2FPEF [54] and European HFA-PEFF [56] studies underscores the importance of AF in the development of HF, and future studies will be needed to further increase our understanding of the role AF may play in development of HFpEF, while they should also focus on the question whether targeting AF could be a potential therapeutic target for treatment of HFpEF.

**Fig. 2:**
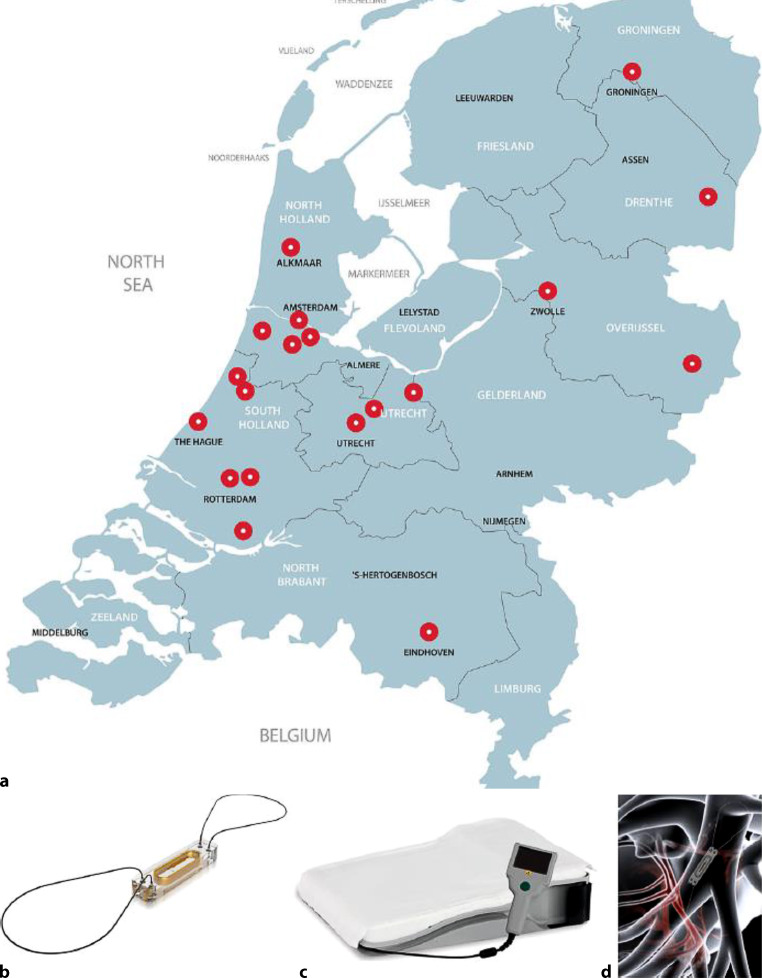
**a** Overview of Dutch sites participating in MONITOR-HF; **b** The CardioMEMS sensor (with permission of Abbott Inc.); **c** The CardioMEMS HF system patient unit including antenna (with permission of Abbott Inc.); **d** Location of the CardioMEMS sensor in the left pulmonary artery (with permission of Abbott Inc)

Development in technology may also help to further shape personalised HF care: in the USA the efficacy and cost-effectiveness of haemodynamic pulmonary artery pressures (e.g. CardioMEMS® device, Abbott) has already been demonstrated. Remote monitoring of pulmonary artery pressures appeared to be a successful method to continuously assess haemodynamic congestion in chronic HF patients, and has resulted in a reduction of 37% in HF hospitalisations [57, 58]. Standard HF care may, however, differ from standard USA HF care and the MONITOR-HF trial aims to evaluate efficacy and cost-effectiveness of haemodynamic monitoring by CardioMEMS in addition to contemporary standard HF care in the Netherlands. This clinical trial, which is supported by healthcare authorities and Abbott, was launched in 2019 and is currently ongoing in 20 Dutch hospitals (Clinical Trial registration number NTR7672) and will help to evaluate whether home monitoring of pulmonary artery pressures will improve HF outcomes for a societal and healthcare perspective (Fig. 2; [59]).

In conclusion, the HF research conducted in the Netherlands covers a broad field and includes a variety of aspects that may contribute to improvement and optimisation of HF prevention, disease management and treatment. We are in an exciting era, where we are moving away from the classical one-size-fits-all approach. The current blend of new pharmacotherapy, technological advances allowing patient-tailored strategies, and improvements in device development will allow even better, more accurate and more personalised treatment regimens for many individuals with HF regardless of its subtype.
